# Are We Ready for NGS HIV Drug Resistance Testing? The Second “Winnipeg Consensus” Symposium

**DOI:** 10.3390/v12060586

**Published:** 2020-05-27

**Authors:** Hezhao Ji, Paul Sandstrom, Roger Paredes, P. Richard Harrigan, Chanson J. Brumme, Santiago Avila Rios, Marc Noguera-Julian, Neil Parkin, Rami Kantor

**Affiliations:** 1 National HIV and Retrovirology Laboratories at JC Wilt Infectious Diseases Research Centre, Public Health Agency of Canada, Winnipeg, MB R3E 3R2, Canada; paul.sandstrom@canada.ca; 2 Department of Medical Microbiology and Infectious Diseases, University of Manitoba, Winnipeg, MB R3E 0J9, Canada; 3 IrsiCaixa AIDS Research Institute, Hospital Germans Trias i Pujol, s/n, 08916 Badalona, Catalonia, Spain; rparedes@irsicaixa.es (R.P.); mnoguera@irsicaixa.es (M.N.-J.); 4 Infectious Diseases Department, Hospital Germans Trias i Pujol, 08916 Badalona, Catalonia, Spain; 5 Division of AIDS, Department of Medicine, University of British Columbia, Vancouver, BC V5Z 1M9, Canada; richard.harrigan@ubc.ca; 6 British Columbia Centre for Excellence in HIV/AIDS, Vancouver, BC V6Z 1Y6, Canada; cbrumme@cfenet.ubc.ca; 7 Division of Infectious Diseases, Department of Medicine, Faculty of Medicine, University of British Columbia, Vancouver, BC V5Z 1M9, Canada; 8 Centre for Research in Infectious Diseases, National Institute of Respiratory Diseases, Mexico City 14080, Mexico; santiago.avila@cieni.org.mx; 9 Chair in AIDS and Related Illnesses, Centre for Health and Social Care Research (CESS), Faculty of Medicine, University of Vic–Central University of Catalonia (UVic–UCC), Can Baumann, Ctra. de Roda, 70, 08500 Vic, Spain; 10 Data First Consulting Inc., Sebastopol, CA 95472, USA; nparkin34@gmail.com; 11 Division of Infectious Diseases, Brown University Alpert Medical School, Providence, RI 02906, USA; rami_kantor@brown.edu

**Keywords:** NGS, HIV drug resistance testing, Winnipeg Consensus, symposium

## Abstract

HIV drug resistance is a major global challenge to successful and sustainable antiretroviral therapy. Next-generation sequencing (NGS)-based HIV drug resistance (HIVDR) assays enable more sensitive and quantitative detection of drug-resistance-associated mutations (DRMs) and outperform Sanger sequencing approaches in detecting lower abundance resistance mutations. While NGS is likely to become the new standard for routine HIVDR testing, many technical and knowledge gaps remain to be resolved before its generalized adoption in regular clinical care, public health, and research. Recognizing this, we conceived and launched an international symposium series on NGS HIVDR, to bring together leading experts in the field to address these issues through in-depth discussions and brainstorming. Following the first symposium in 2018 (Winnipeg, MB Canada, 21–22 February, 2018), a second “Winnipeg Consensus” symposium was held in September 2019 in Winnipeg, Canada, and was focused on external quality assurance strategies for NGS HIVDR assays. In this paper, we summarize this second symposium’s goals and highlights.

## 1. HIV Drug Resistance Is a Global Challenge

With over 40 currently available antiretroviral therapy (ART) drugs, HIV/AIDS has now been successfully converted from a fatal disease into a manageable chronic infection [1,2]. Effective ART not only improves the quality of life of individuals living with HIV, but also minimizes horizontal and vertical HIV transmission contributing to its effective containment on a global scale [3]. However, drug resistance (DR) is a major challenge to treatment success. HIV drug resistance (HIVDR) is facilitated by the virus’ high replication rate, error-prone replication process, and duration of drug selection pressure [4,5,6]. With an average of one error introduced per viral replication cycle, all HIV variants within an infected individual could theoretically be genetically unique forming a highly diversified pool of viruses, or quasispecies. This extraordinary level of diversity creates a large gene pool in which variations, or mutations, may exist at any nucleotide codon in the HIV genome, or any amino acid it encodes [5,7]. ART drugs that are part of a given regimen may effectively eliminate the majority of circulating HIV variants that are sensitive to them. However, under certain circumstances and changing drug selective pressures, HIV variant(s) harboring DR-associated mutations (DRMs) may outgrow and become dominant, due to their selective advantage in the presence of ART drug(s), resulting in resumed replication and ART failure. HIVDR currently constitutes a major obstacle in the maximization of ART benefits at both individual and population levels [8,9].

## 2. Next-Generation Sequencing (NGS) Is an Emerging New Standard for HIVDR Testing

While the cost-effectiveness of HIVDR testing prior to ART initiation or ART regimen switch remains debatable in different contexts [10,11], timely HIVDR detection and ART regimen adjustments play a vital role in the effective clinical management of persons with HIV [12,13]. Conventional genotypic DR assays rely on sequencing of relevant HIV gene fragments using population-based Sanger sequencing (SS) technology to detect known HIV DRMs qualitatively [14]. The resistance profile is then inferred using well-established HIVDR interpretation systems [15]. While SS-based HIVDR testing has been widely applied for decades, some intrinsic constraints limit its ability to quantitatively identify DRMs at frequencies below ~20% of the viral quasispecies [16,17,18]. While integrase inhibitors are being more broadly administered, genotypic resistance analysis on the integrase (IN) gene is often needed. This usually requires a second SS test since it is distant from the protease (PR) and beginning of the reverse transcriptase (RT) genes targeted by routine HIVDR genotyping. Additionally, SS has low data throughput and scalability, which is challenging for laboratories processing large numbers of specimens [19,20].

Propelled largely by the need for affordable genome sequencing, many NGS technologies have been developed and made commercially available since 2005, when the 454 pyrosequencing technology was first launched [21]. Currently, exemplified by the Illumina sequencing-by-synthesis approach, several NGS technologies are available and are being broadly adopted by research and clinical laboratories (Table 1). While sequencing mechanisms vary, all NGS technologies empower massive, parallel, clonal sequencing of input DNA templates without a need for molecular cloning. NGS has become an essential tool in nearly all molecular biology fields [22].

The application of NGS to HIV genotyping began in 2006 when it was primarily used to resolve HIV quasispecies [23]. In 2007, Hoffmann et al. and Wang et al. applied the NGS 454 pyrosequencing platform to HIVDR testing [24,25]. Since then, multiple NGS platforms have been adopted for HIVDR testing by independent laboratories in different contexts worldwide [20,26,27,28,29,30,31,32,33,34,35]. It has been well-demonstrated that NGS outperforms SS for genotypic HIVDR testing in regards to scalability, data throughput, and especially sensitivity for detection of minority resistance variants (MRVs), which may lead to ART failure [16,36]. Additionally, in high-throughput environments where sample batching is feasible, NGS offers improved time efficiency and cost-effectiveness [37]. Meanwhile, although separate SS assays are required to cover the PR + RT and IN gene when needed, sequencing all three genes simultaneously or even beyond can be easily achieved by using longer PCR amplicons or combining different amplicons prior to the fragmentation and tagmentation steps during NGS library preparation [38,39]. While the large number of clonal NGS reads from a single specimen can enable high-resolution analyses of literally all HIVDR variants, the consensus sequences derived from NGS can also be used to mimic SS output in any downstream applications, such as phylogenetic analysis for molecular epidemiology that often requires “one sample, one sequence”. Therefore, NGS HIVDR assays are flexible as they can produce conventional SS-like readouts, while also allowing in-depth quantitation of MRVs.

Although still in its infancy, NGS HIVDR testing holds great promise in enhancing patient management and is likely to become the new genotypic standard [40]. While primarily applied in research settings, recent attempts have been made to incorporate NGS into HIVDR public health surveillance and clinical settings. The Vela Sentosa^®^ SQ HIV-1 Genotyping platform from Vela Diagnostics has been recently approved as the first commercial NGS HIVDR assay for clinical HIVDR testing by regulatory agencies in several settings, including the U.S. Food and Drug Administration (US FDA) [41,42,43]. This Ion Torrent technology-based Sentosa^®^ platform accommodates sequencing of HIVDR relevant genes with sensitivity for MRV detection at 10% when the viral load (VL) is ≥5,000 copies/mL or 20% as VL is lower. However, the high per sample cost (~$400) limits its accessibility for generalized applications for patient care or HIVDR surveillance uses.

## 3. Standardization of NGS HIVDR Testing Is Urgently Required

**Figure 1:**
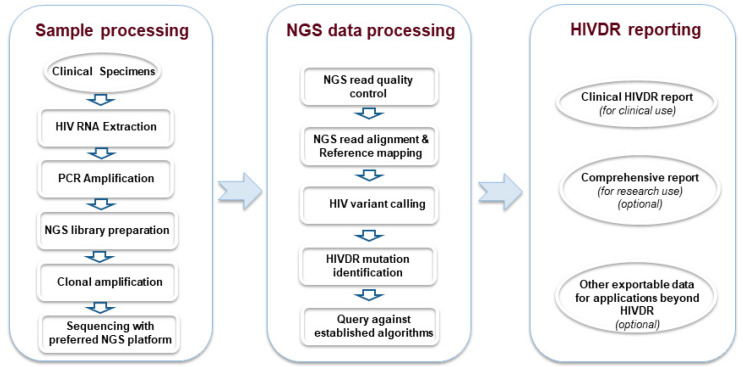
NGS-based HIV drug resistance testing workflow.

NGS HIVDR assays are multiprocedural processes consisting of many quality control (QC) checkpoints to ensure high data quality. They need both well-developed protocols for sample processing in the laboratory, and sophisticated bioinformatics pipelines for automated data processing, unbiased, correct identification of HIV DRMs, and actionable HIVDR reporting (Figure 1). Laboratory procedures involve the extraction of HIV viral RNA, reverse transcription-polymerase chain reaction (RT-PCR) amplification to convert viral RNA into complementary DNA and enrich the target HIV templates, NGS library preparation, and DNA sequencing with the NGS platform of choice. Thus far, many NGS laboratory protocols have been developed and/or published by different groups in varied contexts. Expectedly, significant variations exist among such protocols, especially in regard to in-house developed assays. Likewise, NGS HIVDR data analysis is a complex process involving large volumes of raw data that need to be analyzed for read QC, reference mapping, variant calling, DRM identification, HIVDR interpretation and reporting, and generation of other exportable data (i.e., consensus sequence) for relevant downstream applications. Many such bioinformatics pipelines have also been developed, some publicly available while others proprietary from commercial sources [45]. Despite these advances, most laboratory protocols and pipelines were developed by independent groups with minimal intercommunication among them, and validated using strategies selected by the developers, as no consensus or guidelines for validation are available. These knowledge and technical gaps can inevitably create difficulties for performance assessment of NGS HIVDR protocols, making comparisons among different platforms and methods difficult. Standardization of both molecular and bioinformatics strategies for NGS HIVDR testing is urgently required for the operationalization and general implementation of such assays in routine practice.

## 4. Initiation of an International Symposium Series on NGS HIVDR Testing

Recognizing that many technical and knowledge gaps still exist, in late 2017, we conceived an international symposium series on NGS HIVDR testing. The main aim was to convene leading research scientists, clinicians, bioinformaticians and laboratory experts in the field to communicate and brainstorm relevant strategies and ensure the reliability of NGS HIVDR output for research, public health, and especially patient care needs. The ultimate objective of these symposia is to establish consensus on specific technical aspects associated with NGS for HIVDR testing and to propose best practice recommendations and professional guidelines.

The inaugural NGS HIVDR symposium was held in 2018 in Winnipeg, Canada, and focused on bioinformatics strategies. The developers of four commonly applied, freely available pipelines convened to share their experience in pipeline development, exchange ideas for further improvements and brainstorm the best possible strategies to ensure both the quality and utility of the output data derived from such pipelines. The four pipeline teams included HyDRA from the National Microbiology Laboratory in Canada, PASeq from IrsiCaixa Institute for AIDS Research in Spain, MiCall, from the British Columbia Center for Excellence in HIV/AIDS in Canada, and hivmmer from Brown University in the United States [20,46,47]. The first technical recommendation document for NGS HIVDR data processing was generated in that symposium, now referred to as the first “Winnipeg Consensus” [45]. This document may serve as a prototypic guideline for refinements of existing NGS HIVDR pipelines and for the development of new bioinformatics tools for processing of NGS data from viral pathogens like HIV that harbor significant intra-host genetic diversity. One recent study compared the performance of five different pipelines designed for NGS HIVDR analysis, including HyDRA, MiCall, PASeq, hivmmer and DEEPGEN [48]. Although these pipelines are highly comparable while analyzing mutations at higher abundance, significant discrepancies were observed when variants under the 2% NGS threshold were concerned, largely due to differences in their data management strategies. These findings certainly support the notion that unified NGS HIV data processing strategies are urgently required.

## 5. Are We Ready for NGS HIV Drug Resistance Testing? The Second “Winnipeg Consensus” Symposium

While the bioinformatics strategies for NGS HIVDR are complex, their key functional modules are rather straightforward, and their requirements for ensuring output data quality are relatively definable. In contrast, variations among different NGS assays predominantly result from laboratory procedures through which samples are processed and sequenced, often by different NGS platforms (Figure 1). Such differences could arise from any experimental procedures or, in most cases, a combined consequence from the aggregate workflow. These variations can be further complicated by intra-host (mostly within the same HIV-1 subtype) and inter-host (within the same subtype or among different subtypes) HIV diversity when clinical specimens are examined. Conceivably, all strategies that deal with NGS HIVDR laboratory protocol development, assay validation, or internal and external quality control would never be straightforward but require joint efforts from experts in the field.

The second international symposium on NGS HIVDR testing was held in Winnipeg, Canada, in September 2019. It gathered invited experts in the field from 18 leading institutes in eight different countries. To address gaps described above, the focus of the discussions was on to-be-developed external quality assessment (EQA) strategies for laboratories performing NGS HIVDR assays. In-depth discussions were carried out during the symposium on many different aspects that affect the operationalization of NGS HIVDR tests, especially in clinical laboratory practice. These included: (1) clinical and laboratory advances in NGS HIVDR testing; (2) feasibility and challenges in transitioning from SS towards NGS for HIVDR testing; (3) NGS HIVDR assay validation and internal quality controls strategies, including incorporation of unique molecular identifiers (UMIs); (4) EQA strategies and logistics; (5) development of proficiency testing (PT) panels for NGS HIVDR assays; and (6) laboratory, clinical and implementation considerations that facilitate the general adoption of NGS HIVDR testing; (7) other challenges, such as the implementation status of the first “Winnipeg Consensus” and new bioinformatics challenges identified, especially for accountable variant calling and NGS consensus sequence generation. While reaching a consensus is always the ultimate goal of such a symposium, all delegates acknowledged that more research is still required to better formulate suitable recommendations for NGS HIVDR internal and external quality assurance strategies when laboratory procedures are taken into consideration. Despite this consensus on “no consensus”, important knowledge and technical gaps that hinder the general adoption of such assays in frontline laboratories were better defined. 

## 6. Remaining Challenges for Generalized Implementation of NGS HIVDR Testing

The challenges identified in the symposium that may inform further research to refine existing NGS HIVDR testing methods or support the development of new assays are summarized in Table 2.

## 7. Conclusions

With NGS technology becoming less technically challenging and as the clinical relevance of MRVs is better understood, NGS is trending towards becoming the new standard for HIVDR genotyping. An ideal NGS HIVDR assay would: (1) accommodate the significant intra-host and inter-host HIV diversity; (2) be consistently suitable for all specimen formats (e.g., plasma/serum; dried blood spots or peripheral blood mononuclear cells (PBMCs)) and HIV-1 subtypes at varied VL levels; (3) resolve viral quasispecies with DRMs at high resolution and accuracy; (4) perform well on native HIV templates directly with no requirement for PCR-based library preparation, avoiding related bias and artificial errors; (5) produce long reads covering the entire target gene fragment and enabling HIVDR analysis at the variant rather than mutation level; (6) be low-cost with fast turnaround time, making it suitable for resource-limited settings and/or effective patient care needs; and (7) operate with minimal instrumentation and technical requirements enabling potential point-of-care HIVDR monitoring. Needless to say, meeting these requirements with a single assay is challenging and will require further research and development. The second “Winnipeg Consensus” symposium, highlights of which were presented here, started to address some of the remaining challenges for generalized use of NGS HIVDR testing, hopefully bringing us closer to meet these requirements in the coming years.

## Figures and Tables

**Table 1:** Currently available next-generation sequencing (NGS) sequencing platforms.

Generation of Sequencing Technology ^a^	Manufacturer	Sequencing Mechanism	Error Rates (%)	NGS Platform	Maximum Read Length (Bases) ^b^	Data Throughput (Gigabases/Run)	Estimated Instrument Cost (USD)
2nd generation	Illumina	Sequencing-by-Synthesis	~0.1	iSeq	2 × 150	0.3–1.2	19,900
				MiniSeq	2 × 150	1.7–7.5	50,000
				MiSeq	2 × 300	0.3–15	100,000
				NextSeq	2 × 150	10–120	250,000
				HiSeq	2 × 150	10–1000	650,000
	Thermo Fisher	Ion semiconductor sequencing	~1	PGM	400	0.08–2.0	80,000
				S5	400	0.6–1.5	60,000
				Proton	200	10–15	149,000
	Vela Diagnostics	Ion semiconductor sequencing	~1	Sentosa SQ301	200	0.6–2.0	400,000
3rd generation	Pacific Biosciences	Single-molecule real-time sequencing (no PCR involved)	~13	PacBio RSII	60,000	0.5–1.0	750,000
				Sequel	60,000	5–10	350,000
	Oxford Nanopore Technologies	Single-molecule real-time sequencing (no DNA synthesis involved)	~12	MinIon	100,000+	10–20	1000
				GridIon	100,000+	50–100	2400
				PromethIon	100,000+	480–960	25,000

Notes: ^a^ Considering Sanger sequencing as the 1st generation sequencing technology, the current major NGS platforms are divided into two categories: (1) 2nd generation sequencing technologies which require clonal amplification of the target templates before parallel clonal sequencing steps; (2) 3rd generation sequencing technologies which feature single-molecule, real-time sequencing with no requirement for pre-PCR amplifications for template enrichment, or sequencing of native DNA templates in real-time involving no DNA synthesis [22]. ^b^ “2×” indicates paired-end sequencing available. This table was adapted from reference [44].

**Table 2:** Challenges for the generalized application of NGS-based HIV drug resistance (HIVDR) testing.

Challenges	Operational Needs and Current Status	Steps Moving Forward
Lack of appropriate reference materials for EQA and PT	▪ NGS HIVDR testing features for sensitive detection of DRMs both qualitatively and quantitatively. ▪ Well-designed and fully characterized reference materials that closely approximate clinical specimens, with known DRMs and their exact frequencies, are essential for assay validation and quality assurance.	▪ PT specimens (“wet panels”) that approximate clinical specimens of different formats with characteristic HIV-1 diversity, varied VLs, an array of major subtypes and recombinant forms, and DRMs at varied abundance are required; ▪ Well-characterized reference datasets (“dry panels”) consisting of authentic NGS data and in silico data files that resemble raw NGS output data derived from varied NGS platforms using specimens of different subtypes and/or containing different levels of DRMs are in need.
Lack of protocols that work consistently, without sampling bias, for different HIV-1 subtypes, specimen formats or VLs	▪ Some NGS HIVDR testing protocols with broad HIV-1 subtype coverage and/or high accuracy have been reported. The Vela Sentosa^®^ platform has obtained approvals from several regulatory agencies, including the US FDA, for clinical HIVDR testing. ▪ Most NGS HIVDR protocols involve rounds of PCR amplification in NGS library preparation. Probe-capture-based NGS methods, such as veSEQ-HIV, require minimal PCR steps and may help to reduce PCR-introduced sampling bias [49]. ▪ Validations of most existing NGS HIVDR assays were based on comparisons with SS-based tests for concordance in DRM detection; the sensitivity for MRV detection was largely determined using pre-mixed plasmids or molecular HIV clones.	▪ The representativeness of NGS reads for the intra-host viral diversity, or the comprehensiveness of an NGS HIVDR assay in resolving HIV quasispecies should be better determined. ▪ Strategies that better quantify the original input templates into the NGS workflow are imperative for improved characterization of an NGS HIVDR assay and ensuring its accountability for HIV DRM analysis, especially when MRVs are concerned. ▪ Technologies such as UMIs are essential for boosting the accuracy of HIV DRM detection. Incorporation of such technologies in an NGS assay is highly recommended when exact DRM frequency readouts are needed [31,50,51].
Lack of simplified and cost-effective assays suitable for resource-limited settings and/or point-of-care use	▪ Transitioning from SS- to NGS-based HIVDR assays requires not only a “standardized” methodology with proven performance, but also improved accessibility to the needed infrastructure. ▪ While more NGS platform options are currently available and costs are falling, the demanding instrumentation requirements, high cost of consumables and limited access to technical support involved in NGS remain major barriers that hinder its general adoption in HIVDR laboratories. ▪ The prohibitive per sample cost and long turnaround time if relying on batched tests for cost reduction, limit applications of such assays for routine patient care.	▪ Cost-effective NGS HIVDR tests with a fast turnaround time suitable for individualized sample testing are urgently needed for better implementation of such technologies in resource-limited settings and/or for patient care applications [37]. ▪ Novel approaches that allow more aggressive multiplexing and greater scalability of an NGS HIVDR assay will improve cost-effectiveness when performing centralized, batched specimen testing for clinical monitoring or surveillance applications. ▪ New and more affordable NGS sequencing technologies are beginning to emerge, such as Oxford Nanopore technology (https://nanoporetech.com). As they mature, such techniques may help to turn point-of-care NGS HIVDR testing into reality.
Lack of unified assay validation and internal quality control (IQC) strategies	▪ Well-defined and widely accepted assay validation and IQC strategies are essential for nucleic acid tests, including NGS HIVDR assays. ▪ NGS HIVDR assays are multiprocedural tests aiming to resolve the intrinsic diversity of HIV quasispecies in specimens and identify DRMs quantitatively, as compared to SS methods that are qualitative or semi-quantitative with the appropriate tools. ▪ NGS HIVDR assays have many unique characteristics rendering conventional SS-based assay validation, characterization and IQC strategies insufficient.	▪ Suitable assay validation and characterization parameters should be defined, and meaningful cut-offs or reference values for them should be established. ▪ New assay performance assessment platforms that incorporate the newly defined parameters and standards are required for effective NGS HIVDR assay characterization within the laboratory and performance evaluation of such assays for accreditation purposes by regulatory agencies. This is especially critical as NGS HIVDR testing moves towards clinical use. ▪ The development of well-characterized reference materials with predetermined ground truth about the exact frequencies of all present HIV DRMs is urgently required.
Lack of effective EQA strategies that enable objective laboratory performance assessment	▪ EQA assesses the capacity of a laboratory in effectively conducting a designated assay. ▪ EQA plays a vital role in ensuring the quality of data from laboratories offering SS-based HIVDR testing. ▪ Compared to SS-based qualitative HIVDR tests, NGS-based quantitative HIVDR assays are far more complex. NGS consensus sequence-based EQA analysis by, direct application of strategies designed for SS methods, oversimplifies the intrinsic complexity of NGS HIVDR data output [52,53]. ▪ EQA strategies and EQA programs that satisfy the specific needs for NGS HIVDR assays remain to be developed.	▪ Innovative parameters that enable meaningful and objective assessment of laboratory performance in conducting NGS-based HIVDR assays should be established, validated and operational “standards” for such metrics should be formulated; ▪ Rational and practical EQA scoring strategies that implement newly developed EQA parameters should be developed before generalized implementation of NGS HIVDR assays [54,55]. ▪ Logistics schemes that enable or facilitate such EQA strategies need to be developed. ▪ An operational and sustainable EQA program that implements the above-described strategies. ▪ Standardized training guideline(s) and assisted troubleshooting actions, offered via EQA programs, will help to improve staff capacity in properly conducting such assays.
Short NGS reads that hinder quasispecies reconstruction and downstream cluster analysis	▪ As compared to SS which generates “one sequence per specimen”, NGS assays produce a wealth of sequence data that enables varied downstream analyses. ▪ The majority of NGS HIVDR assays rely on Illumina or Ion Torrent technologies, the maximum read lengths from which are 600 nucleotides. ▪ While the length of individual sequences has little effect on the identification of HIV DRMs, short NGS read lengths and high genetic diversity makes it difficult to analyze HIV quasispecies at the haplotype or variant level using existing haplotype callers [56]. ▪ While HIV *pol* gene sequencing largely serves the needs for HIVDR genotyping, such sequencing data is often also applied in molecular epidemiology, such as cluster analysis. Thus far, NGS-based cluster analyses are largely based upon (1) NGS consensus sequences that simply mimic SS sequences; (2) reconstructed viral variants using quasispecies reconstruction tools that often perform poorly on HIV NGS data [56,57].	▪ Sophisticated bioinformatics tools that enable effective haplotype or variant constructions from short NGS reads of high genetic diversity are still required for identifying individual variants in viral quasispecies or resolving the authentic combinations (“linkage”) of HIV DRMs within an HIV variant. ▪ An NGS assay capable of producing longer reads, or full-length viral genome if possible, would enable a better understanding of HIV diversity within infected individuals. Notably, both Nanopore and Pacific Bioscience technologies have the capacity to produce full-genome HIV sequences, but high sequence error rates and demanding template requirements limit their applicability in HIV sequencing currently. ▪ While further refinements are made to existing haplotype or quasispecies reconstruction tools or as new tools are developed for improved performance on NGS data of high genetic diversity, novel bioinformatics approaches that take full advantage of the wealth of NGS data information are desired. Tools that enable cluster analysis using the clonal NGS reads from different specimens directly (“reads vs. reads”), or examine the evolutional relatedness by directly estimating the genetic distance among quasispecies (“profile vs. profile”) [58], would be beneficial.
Tools for improved bioinformatic data processing and HIVDR interpretation	▪ Unified HIVDR interpretation and reporting criteria are required for standardized NGS HIVDR tests to minimize the subjectivity of the data management procedures. ▪ The first “Winnipeg Consensus” outlined essential bioinformatics strategies that ensure reliable and actionable output data from NGS HIVDR assays. It also recommended a standard Amino Acid Variant Format (AAVF) to report mutations from NGS-based genotyping to facilitate the integration of data from varied sources [45]. ▪ The Stanford University HIV drug resistance database team has a new web tool (HIVdb-NGS) currently being tested. It accepts NGS codon frequency files (i.e., AAVF) and provides HIVDR interpretation and reports based on Stanford HIVdb algorithms. The web tool also profiles numbers of unusual and signature human APOBEC-mediated mutations in the HIV *pol* gene, the majority of which are not associated with DRMs, at different frequency thresholds to help users better identify an appropriate mutation detection cut-off. (https://hivdb.stanford.edu/page/hivdb-ngs-release-notes/).	▪ While most recommendations from the “Winnipeg Consensus” have been implemented in some freely available pipelines (e.g., HyDRA, PASeq, MiCall), further efforts are required to maximize the benefit of this consensus and unify the bioinformatic analysis strategies, especially those for HIV DRM calling/reporting and NGS consensus generation [45]. ▪ The new HIVdb-NGS tool may serve as a platform to standardize NGS HIVDR interpretation and reporting as it is compatible with any pipeline that generates AAVF files, regardless of the front-end data processing steps. Moreover, it moves relevant analyses online and simplifies data processing steps for HIVDR interpretation from NGS data, enabling access for laboratories that lack resources and highly qualified personnel to develop their own bioinformatics pipeline. ▪ Any new bioinformatics tool that allows automated NGS data analysis, HIVDR interpretation, and/or clinical HIVDR reporting would benefit from adoption of such technology. ▪ Strategies that enable seamless connections between existing NGS data analysis pipelines and tools like HIVdb-NGS, or new platforms incorporating all their functionalities would be beneficial.
The clinical relevance of NGS-identified MRVs remains to be better defined	▪ As compared to SS-based methods, NGS HIVDR assays are credited with enhanced sensitivity for detecting all HIV variations, including MRVs at population frequencies lower than ~20%. ▪ Despite their lower abundance, increasing evidence suggests that MRVs may also lead to ART failure [16,18,36]. However, the exact abundance cut-off at which MRVs become clinically relevant remains to be better defined, recognizing that these may depend on factors like specific DRMs and/or mutation loads [36,59,60].	▪ Better understanding of the clinical relevance of MRVs via scaled clinical trials will help refine NGS HIVDR interpretation and improve strategies for clinical patient care [61]. ▪ The original HIV template input copy number and the sampling evenness of different variants from the initial viral quasispecies directly affect the capacity of an NGS assay to resolve the abundance of MRVs in the original specimen. Precautions should be taken when reporting the exact frequencies of MRVs from NGS HIVDR assays in settings when the initial input HIV template copy number is not traceable.
